# Influence of *Murraya koenigii* extract on diabetes induced rat brain aging

**DOI:** 10.25122/jml-2022-0151

**Published:** 2023-02

**Authors:** Lakshmi Bhupatiraju, Krupavaram Bethala, Khang Wen Goh, Jagjit Singh Dhaliwal, Tan Ching Siang, Shasidharan Menon, Bamavv Menon, Kishore Babu Anchu, Siok Yee Chan, Long Chiau Ming, Abdullah Khan

**Affiliations:** 1 Department of Pharmacology, School of Allied Health Sciences, Malla Reddy University, Hyderabad, Telangana, India; 2 Department of Pharmacology, School of Pharmacy, KPJ Healthcare University College, Nilai, Malaysia; 3 Faculty of Data Science and Information Technology, INTI International University, Nilai, Malaysia; 4 PAPRSB Institute of Health Sciences, Universiti Brunei Darussalam, Gadong, Brunei Darussalam; 5 School of Pharmaceutical Science, Universiti Sains Malaysia, Minden, Malaysia; 6 School of Medicine and Life Sciences, Sunway University, Sunway City, Malaysia; 7 Faculty of Pharmacy, Quest International University, Ipoh, Malaysia

**Keywords:** *Murraya koenigii*, diabetes, dementia, neurodegeneration, oxidative stress, anti-oxidant, nootropic, AChE – acetyl cholinesterase, AD – Alzheimer’s disease, DTNB – dithiobis nitrobenzoic acid, EDTA – ethylene diamine-tetra-acetic acid, i.p. – intra peritoneal, kg – kilogram, mg – milligram, ml – milliliter, p.o. – per oral, w/v – weight/volume, w/w – weight/weight

## Abstract

Food supplements are used to improve cognitive functions in age-related dementia. This study was designed to determine the *Murraya koenigii* leaves’ effect on Alloxan-induced cognitive impairment in diabetic rats and the contents of oxidative stress biomarkers, catalase, reduced glutathione, and glutathione reductase in brain tissue homogenates. Wistar rats were divided into seven groups (six rats per group). Group I received saline water (1 ml, p.o.), Diabetes was induced in Groups II–VII with Alloxan (120 mg/kg/p.o). Group III was provided with Donepezil HCl (2.5 mg/kg/p.o.), Group IV, V, VI, and VII with *Murraya koenigii* ethanol extract (200 and 400 mg/kg/p.o.) and aqueous extract (200 and 400 mg/kg/p.o.), respectively, for 30 days. Behavior, acetylcholinesterase (AChE) activity, oxidative stress status, and histopathological features were determined in the hippocampus and cerebral cortex. Administration of *Murraya koenigii* ethanolic and aqueous extracts significantly (P<0.05, P<0.001) increased the number of holes crossed by rats from one chamber to another. There was an increase in the (1) latency to reach the solid platform, (2) number of squares traveled by rats on the 30^th^ day, and (3) percentage of spontaneous alternation behavior compared to the control group. Administration for successive days markedly decreased AChE activity (P<0.05), decreased TBARS level, and increased catalase, GSH, and GR levels. Murayya koenigii could be a promising food supplement for people with dementia. However, more research into sub-chronic toxicity and pharmacokinetic and pharmacodynamics interactions is essential.

## INTRODUCTION

Diabetes mellitus (DM) is a very common chronic disease where there is a gradual deterioration of organs in the body and is associated with microvascular and macrovascular complications. One of the later complications is cognitive decline and dementia, representing a serious problem in the elderly population. Cognitive impairment in diabetic patients requires immediate research solutions. It should also be addressed along with microvascular complications such as neuropathy, nephropathy, retinopathy, and cardiovascular complications.

Alloxan is a diabetogenic agent used in diabetes research to induce insulin reduction. It acts by aggregating the pancreatic β-cells via the Glut2 glucose transporter and demolishes them through reactive oxygen species (ROS) and free radicals mechanisms [1]. Oxidative stress plays an important role in the enhancement of diabetes complications, including learning and memory impairments, as a result of the increased generation of free radicals and diminished antioxidant defenses [2]. These free radicals lead to increased neuronal death in several brain areas, including the hippocampus, and DNA damage, through protein oxidation and peroxidation of membrane lipids [3].

Since ancient times medicinal plants and their chemical constituents have been widely used to cure or mitigate diseases. India is well-known for its vast medicinal plant biodiversity. *Murraya koenigii*, for example, includes several bioactive components [4], as a result based on which the plant has been demonstrated to be medicinally essential, but it has received little or no attention from scientists. *Murraya koenigii* (L.) Spreng. (Family: Rutaceae) is known as Curry Leaf in English, Mitha Neem or Kadi Patta in Hindi, Surabhinimba in Sanskrit, and Karuveppilei in Tamil. It is commonly utilized as a spice and condiment in India and has been demonstrated to have natural healing properties [5]. Plant-derived (phyto) carbazole alkaloids are an important class of compounds present in the family of Rutaceae (*Genera Murraya, Clausena, Glycosmis, Micromelum*, and *Zanthoxylum*). Due to several significant biological activities, such as antitumor, antibacterial, antiviral, antidiabetic, anti-HIV, and neuroprotective activities of the parent skeleton (3-methylcarbazole), carbazole alkaloids are recognized as an important class of potential therapeutic agents.

Since ancient times roots and leaves of *Murraya koenigii* are traditionally used to treat various GIT disorders. They are known to promote appetite, treat nausea, and control flatulence, diarrhea, and dysentery. Moreover, *Murraya koenigii* is also used to relieve pain, reduce fever, cancer, and hemorrhoids, and acts as an antidote against animal bites [5]. The *Murraya koenigii* leaf extracts are used to treat diabetes. Phytoconstituents like carbazole alkaloids, glycosides, flavonoids, minerals, and volatile oil are found in this plant [6]. *Murraya koenigii* can be used directly or in a variety of forms, including extracts and essential oils. The presence of active constituents such as bismahanine, murrayanine, murrayafoline-A, bi-koeniquinone-A, bismurrayaquinone, mukoenine-A, mukoenine-B, mukoenine-C, murrastifoline, Murrayazolinol, murrayacine, murrayazolidine, murrayazoline, mahanimbine, girinimbine, koenioline, xynthyletin, koenigine-Quinone A and koenigine-Quinone B make it highly valuable [7-10].

Preliminary studies reported that the plant has anti-diabetic and neuroprotective activities [11, 12]. Based on our previous preliminary studies, *Murraya koenigii* (L.) Spreng leaf extracts have shown significant neuroprotective activity in the aluminum-induced cognitive deficits model [13]. Hence, we have designed the current study to evaluate the cognitive enhancing potential of *Murraya koenigii* (L.) Spreng leaf extracts in Alloxan-induced cognitive decline and brain tissue oxidative stress via behavioral and biochemical study in rats.

## MATERIAL AND METHODS

### Drugs and chemicals

All the chemicals and reagents used in the study were of analytical grade, respectively Donepezil hydrochloride (Incepta Pharmaceuticals Ltd. Dhaka, Bangladesh) and Alloxan monohydrate (Explicit Chemicals, Pvt. Ltd. Pune, India). A blood glucometer (Bayer Healthcare, India) was used.

### Plant collection & identification

Murrayya koengii leaves were procured from the Maisammaguda area, Hyderabad, and the sample was authenticated at the Botanical department, Osmania University, Hyderabad, and voucher specimen: MK0152 was deposited.

### Preparation of ethanolic extract

The collected plant material was air-dried and made into a coarse powder using a grinder. 100 g of leaf powder was filled in soxhlet apparatus and subjected to ethanol (95%) extraction. After 72 hours, the mixture was filtered, concentrated under reduced pressure to obtain a semisolid extract, and stored in an air-tight flask in the refrigerator for later use.

### Preparation of aqueous extract

The dried plant material of *Murraya koenigii* was powdered. Chloroform and water were added in 1:9 ratios into the conical flask. The powder was poured into the flask and subjected to frequent agitation at 10 minutes intervals for 48 hours. Later the powder was filtered with the help of a muslin cloth. Liquid filtrates are concentrated and evaporated to dryness using a rotary evaporator under reduced pressure to get the crude extract (10.57 g) stored in the refrigerator for further evaluation. The extracts were subjected to preliminary phytochemical identification by the standard procedures given in “Practical Pharmacognosy” by C.K. Kokate. The doses of donepezil and alloxan were selected according to the literature review [14-16]. The doses of *Murraya koenigii* extracts were selected according to our previous studies [17, 18].

### Selection and maintenance of animals

Adult Wistar Albino rats of either sex, weighing between 150 and 250 grams, were used. The animals were kept in conventional polypropylene cages at room temperature and given ad libitum access to food and water.

### Induction of experimental Diabetes Mellitus and experimental design

Rats fasted overnight with free access to water before the experiment. Alloxan monohydrate (120 mg/kg b.w. in 0.9 % cold normal saline) was given with a single intraperitoneal injection. The rats were given a 5% glucose solution to prevent hypoglycemia [19].

Forty-two Wistar Albino rats weighing between 200gms-250gms were chosen for the study. Animals were divided into seven groups of six animals each. Rats of groups II-VII were injected with alloxan to induce diabetes. Blood glucose levels were analyzed using a digital glucometer, and the blood sample was collected from the animal's tail. Animals with blood glucose levels< 200 mg/dl were considered hypoglycemic. Groups III-VII rats were continued with respective treatments:

- Group 1: The control group received normal saline water;
- Group 2: Alloxan monohydrate (120mg/kg, i.p.) was administered to rats;
- Group 3: Alloxan monohydrate (120mg/kg, i.p.) + Donepezil hydrochloride (2.5mg/kg, p.o.);
- Group 4: Alloxan monohydrate (120mg/kg, i.p.) + ethanolic extract of *Murraya koenigii* (200mg/kg, p.o.);
- Group 5: Alloxan monohydrate (120mg/kg, i.p.) + ethanolic extract of *Murraya koenigii* (400mg/kg, p.o.);
- Group 6: Alloxan monohydrate (120mg/kg, i.p.) + aqueous extract of *Murraya koenigii* (200mg/kg, p.o.);
- Group 7: Alloxan monohydrate (120mg/kg, i.p.) + aqueous extract of *Murraya koenigii* (400mg/kg, p.o.).

The treatment duration was for 30 days. All the doses were given through the oral route by oral lavage. After completion of respective treatments for 30 days, the rats were subjected to evaluation of the influence of *Murraya koenigii* ethanolic and aqueous extracts on behavioral parameters for assessment of cognitive decline induced by diabetes.

### Evaluation of behavioral parameters

#### Hole cross test

The experiment was carried out with a wooden box (30cm×20cm×1cm). A fixed partition in the center of the box and a hole (3 cm in diameter) were available at eight of 7.5 cm from the base. On the day of the experiment, every rat was placed on one side of the apparatus; spontaneous movement from one chamber to another through the hole was observed for 3 minutes on the 28th day [20].

#### Passive Avoidance test

The experiment was performed in an apparatus that consisted of one dark and one light chamber and was divided by a wall. On day one, i.e., acquisition trail, every rat was first placed in the light chamber and then into a dark chamber, and an electric shock (40V, 0.5mA for 1 second) was delivered to the feet of the rat through the grid floor. After the training session, the rat was immediately returned to the cage [21]. On day two, the animal was placed again in the light chamber and the time taken to access the dark chamber was recorded as step-through latency. If the animal did not enter the dark chamber within a 5-minutes test period, the test was terminated, and the step-through latency was recorded as 300 seconds.

#### Morris water maze test

The equipment consisted of a circular tank, and a platform was placed 2 cm below the water level. Milk was added to make the water opaque so that the immersed platform was not visible. The tank was divided into four quadrants. During the training sessions, the animal was trained to find the submerged platform. On the day of the experiment, the latency from immersion into the pool to escape onto the hidden platform (maximum duration of 90 seconds) was recorded [22].

#### Y-maze test

The y-maze test is commonly employed to assess short-term memory in rats. The equipment consisted of three uniform arms 40 cm long, 12 cm high, 5 cm wide at the bottom, and 10 cm wide at the top, and the arms were separated by 120°. It also consisted of a central equilateral triangle area for the rat to enter into any of the arms. On the 30th day, each rat was placed in one of the arms and was permitted to explore for 8 minutes. Spontaneous alternation behavior was noted, i.e., entry into all three arms on successive choices. Then the sequence and number of arm entries were recorded manually. The percentage of spontaneous alternation behavior was calculated according to the following formula [23]:


$$Spontaneous\;alteration\;behavior\;\left(\%\right)=Na/Nabc\;–\;2\;\times \;100\%$$

Na is the number of alternations, and Nabc is the total number of arm entries.

#### Open field test

The apparatus consisted of a wooden box, and the floor was divided into 25 (5x5) squares. The rats were placed into one corner of the open field chamber, and their behavior was observed for 5 minutes. The following observations were made- a) the number of squares explored, the number of central nine squares and peripheral 16 adjacent squares to the wall explored, b) the number of grooming, and c) the number of rearing [24].

#### Estimation of oxidative stress biomarkers

At the end of the experiment, animals were euthanized on the 30th day by CO2 inhalation through the euthanasia chamber and then the brain was isolated carefully and washed with saline solution. The brain was then minced into small pieces and homogenized with phosphate buffer (0.1M, pH-7.4) using a tissue homogenizer to obtain 1:9 (w/v) (10%) whole homogenate. Then the homogenates were centrifuged at 4000 rpm at 4°C for 20 minutes using Remi cool centrifuge, and the resultant supernatant was collected and stored for estimation of tissue antioxidant parameters like malondialdehyde [25], glutathione reductase, and reduced glutathione [26] catalase [27] and brain acetylcholinesterase activity [28]. The brain was excised after the experiment and fixed in formalin (10% v/v). The tissue was processed, sections were cut, and the slides were prepared and stained with Haematoxylin and Eosin, examined under high power microscope (100x), (400x), and photomicrographs were taken.

#### Statistical analysis

Values are expressed as mean ± SEM, using t-test, the intergroup variation between various groups was conducted by graph pad Prism software & data were analyzed by one-way analysis of variance (ANOVA), and P<0.05 was considered to be statistically significant.

## RESULTS

### Effect of *Murraya koenigii* extracts on hole cross test in Alloxan-induced diabetic rats

**Figure 1:**
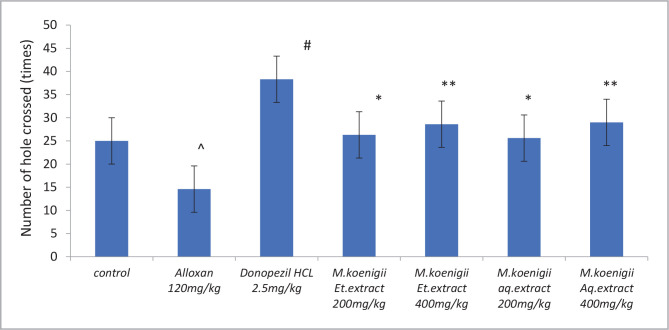
Effect of Ethanol and Aqueous extract of *Murraya koenigii* leaves in Alloxan induced cognitive impairment in diabetic rats using hole cross test. Values are given as mean±SEM, n=6. Using t-test, variations between groups were done by graph pad Prism software, & Data was assessed by one-way ANOVA ^P<0.0001 vs. control group, ^#^p<0.001, *p<0.05, **p<0.001 vs. Alloxan-induced diabetic group.

Alloxan-induced diabetic group exhibited a significant decrease (^P<0.0001) in the number of holes crossed from chamber to chamber in comparison to the control group. *Murraya koenigii* and donepezil-treated groups also restored the Alloxan-induced cognitive impairment in rats (*P<0.0001) compared with the diabetic group, which signifies progress in spatial memory and learning (Figure 1).

### Effect of *Murraya koenigii* extracts on passive avoidance test in Alloxan-induced diabetic rats

**Figure 2:**
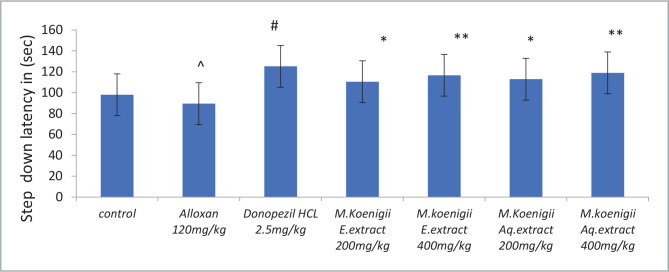
Effect of Ethanol and Aqueous extract of *Murraya koenigii* leaves on a step down latency in Alloxan-induced cognitive impairment in diabetic rats using Passive avoidance test. Values are given as mean±SEM, n=6. Using t-test variations between groups were done by graph pad Prism software, & Data was assessed by one-way ANOVA ^P<0.0001 vs. control group, ^#^p< 0.001, *p<0.05, **p<0.001 vs. Alloxan-induced diabetic group.

Alloxan induced diabetic group showed a significant decrease (^P<0.0001) in the time taken to step down from the solid platform onto the grid floor when compared to the control group. *Murraya koenigii* extract-treated groups showed an increase (*P<0.0001 and **P<0.0001) in the time latency when compared with the Alloxan-treated group (Figure 2).

### Effect of *Murraya koenigii* extracts in Morris water maze test in Alloxan-induced diabetic rats

Alloxan-induced diabetic group showed a significant increase (^P<0.0007) in the time taken to reach the solid platform when compared to the control group. *Murraya koenigii*-treated groups showed a dose-dependent decrease (*P<0.0001, **P<0.0001) as compared with the Alloxan-treated group (Table 1).

**Table 1:** Effect of Ethanol and Aqueous extract of *Murraya koenigii* leaves on latency in Alloxan induced cognitive impairment in diabetic rats using Morris water maze test.

Treatments	Quadrant 1 (Latency in sec)	Quadrant 2 (Latency in sec)	Quadrant 3 (Latency in sec)	Quadrant 4 (Latency in sec)
**Control**	22.4±0.37	25.6±0.50	28.3±0.38	26.1±0.30
**Alloxan 120 mg/kg, i.p**	14.2±0.35^	15.4±0.50^	18.2±0.58^	16.2±0.58^
**Donepezil HCL 2.5 mg/kg, p.o.**	45.1±0.64^#^	42.6±0.34^#^	45.3±0.41^#^	49.7±0.41^#^
***M. koenigii* Et.extract 200 mg/kg, p.o.**	35.7±0.67*	37.6±0.69*	39.3±0.40*	40±0.40**
***M. koenigii* Et.extract 400 mg/kg, p.o.**	37.2±0.511*	36.4±0.41*	40.2±0.15*	41.2±0.15**
***M. koenigii* Aq.extract 200 mg/kg, p.o.**	34.6±0.73*	36.7±0.32*	33.2±0.39*	36.9±0.26*
***M. koenigii* Aq.extract 400 mg/kg, p.o.**	38.2±0.50*	37.5±0.45*	39.6±0.62*	36.9±0.54*

Values are given as mean±SEM, n=6. Using t-test variations between groups was done by graph pad Prism software, & Data was assessed by one-way ANOVA ^p<0.0001 vs. control group, #p<0.001, *p<0.05, **p<0.001 vs. Alloxan-induced diabetic group.

### Effect of *Murraya koenigii* extracts on Y-maze test in Alloxan-induced diabetic rats

In *Murraya koenigii* (P<0.05, P<0.01) and donepezil (P<0.001) treated groups, there was an increase in the percentage of spontaneous alternation behavior as compared with alloxan-induced diabetic rats. This signifies that there is an enhancement in spatial short-term memory and learning (Table 2).

**Table 2:** Effect of Ethanol and Aqueous extract of *Murraya koenigii* leaves on Spontaneous alterations in Alloxan-induced cognitive impairment in diabetic rats using Y-maze test.

Treatments	% Spontaneous alterations
**Control**	69.15±0.40
**Alloxan 120 mg/kg (i.p)**	35.24±0.31^
**Donepezil HCL 2.5 mg/kg (oral)**	70.01±0.42^#^
***M. koenigii* Et.extract 200 mg/kg (oral)**	42.32±0.35**
***M. koenigii* Et.extract 400 mg/kg (oral)**	75.10±0.56**
***M. koenigii* Aq.extract 200 mg/kg (oral)**	47.05±0.35*
***M. koenigii* Aq.extract 400 mg/kg (oral)**	74.82±0.55*

Values are given as mean±SEM n=6. Using t-test, variations between groups were done by graph pad Prism software, & Data was assessed by one-way ANOVA ^p<0.0001 vs. control group, #p<0.001, *p<0.05, **p<0.001 vs. Alloxan induced diabetic group.

### Effect of *Murraya koenigii* extracts on an open field test in Alloxan-induced diabetic rats

Alloxan-induced diabetic group showed a significant decrease (^P<0.0001) in the activities and number of squares explored when compared to the control group. Donepezil-treated rats showed a significant (P < 0.001) increase as compared to the Alloxan-induced diabetic rats. *Murraya koenigii* treated groups showed a significant increase (*P<0.001 and **P<0.0001) in the activities and squares explored compared to Alloxan treated group (Table 3).

**Table 3:** Effect of Ethanol and Aqueous extract of *Murraya koenigii* leaves on behavioral parameters in Alloxan induced cognitive impairment in diabetic rats using Open field test

Treatments	No. of Central squares crossed	No. of Peripheral squares crossed	No. of Groomings	No. of Rearings
**Control**	103.2±1.02	107.5±0.35	105.2±0.32	106.6±0.53
**Alloxan treated 120 mg/kg**	80.2±0.89^	78.6±0.71^	70.5±0.27^	63.2±0.37^
**Donepezil HCL 2.5 mg/kg, p.o.**	125.5±0.72^#^	130.6±0.33^#^	140.3±0.24^#^	138.2±0.35^#^
***M. koenigii* Et.extract 200 mg/kg,p.o**	105.2±0.66*	109±0.30*	113.2±0.75*	115.6±0.68*
***M. koenigii* Et.extract 400 mg/kg, p.o**	107±0.6**	117±0.60**	119.3±0.51**	120.2±0.54**
***M. koenigii* Aq.extract 200 mg/kg, p.o.**	103.9±0.21*	105.6±0.63*	115.6±0.63*	119.6±0.82*
***M. koenigii* Aq.extract 400 mg/kg, p.o.**	108±0.52**	119±0.56**	120.5±0.34**	123.2±0.49**

Values are given as mean±SEM, n=6. Using t-test variations between groups was done by graph pad Prism software, & Data was assessed by one-way ANOVA ^p<0.0001 vs. control group, ^#^p<0.001, *p<0.05, **p<0.001 vs. Alloxan-induced diabetic group.

### Effect of *Murraya koenigii* extracts on the rats’ tissue antioxidant parameters

The oxidative lipid damage was high in the Alloxan-induced diabetic rats, as indicated by significantly increased MDA levels (P<0.0001) as compared with the *Murraya koenigii* extracts treated group. Results presented here indicate that Alloxan-induced cognitive decline was significantly attenuated by *Murraya koenigii* extracts. There was a significant decrease in catalase GSH and GR levels in Alloxan-treated groups (P<0.0001) in comparison to normal control rats. *Murraya koenigii* ethanol and aqueous 200 and 400mg/kg treated groups showed a significant increase (P<0.0001) compared to Alloxan-treated groups (Table 4).

**Table 4:** Effect of Ethanol and Aqueous extract of *Murraya koenigii* leaves on antioxidant parameters in Alloxan induced cognitive impairment in diabetic rats.

Treatments	Catalase (nmoles/mg protein)	MDA (µmoles/mg protein)	GSH (µmoles/mg protein)	GR (µmoles/mg protein)
**Control**	42.9±0.24	42.9±0.32	41.2±0.36	23.0±0.54
**Alloxan 120 mg/kg (i.p)**	31.6±0.42^	79.60.69±^	22.6±0.47^	16.4±0.37^
**Donepezil HCL 2.5 mg/kg (p.o.)**	41.6±0.34^#^	38.0±0.31^#^	54.2±0.27^#^	20.4±0.40^#^
***M. koenigii* Et.extract 200 mg/kg (p.o)**	35.9±0.92**	64.6±0.31*	40±0.44**	18.1±0.38*
***M. koenigii* Et.extract 400 mg/kg (p.o)**	41.0±0.44*	43.9±0.61**	53.5±0.42*	21.2±0.32*
***M. koenigii* Aq.extract 200 mg/kg (p.o)**	33.3±0.38*	53.6±0.18*	31.4±0.29*	17.4±0.28*
***M. koenigii* Aq.extract 400 mg/kg (p.o)**	45.6±0.45*	45.9±0.59**	52.6±0.49*	25.9±0.32*

Values are given as mean±SEM, n=6. Using t-test variations between groups was done by graph pad Prism software, & Data was assessed by one-way ANOVA ^p<0.0001 vs. control group, ^#^p<0.001, *p<0.05, **p<0.001 vs. Alloxan-induced diabetic group.

### Effects of *Murraya koenigii* extracts on Acetylcholinesterase Activity (AChE) on rat brain

**Figure 3:**
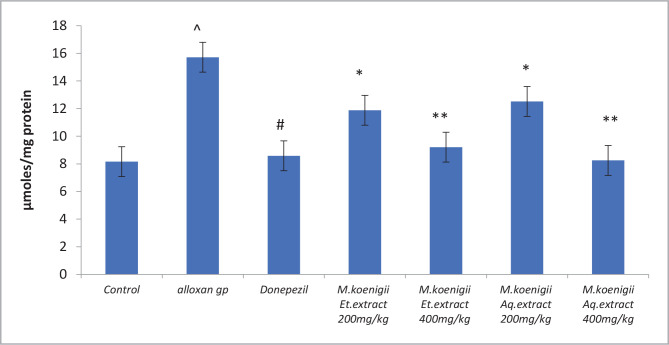
Effect of Ethanol and Aqueous extract of *Murraya koenigii* leaves on Acetylcholinesterase activity in Alloxan induced cognitive impairment in diabetic rats. Values are given as mean±SEM, n=6. Using t-test variations between groups was done by graph pad Prism software, & Data was assessed by one-way ANOVA ^P<0.0001 vs. control group, ^#^p< 0.001 *p<0.05, **p<0.001 vs. Alloxan-induced diabetic group.

Alloxan-induced cognitive decline was evidenced by a significant increase in AChE activity (p < 0.001) in the hippocampus and cortex region (p < 0.01). Treatment with *Murraya koenigii* extracts significantly prevented the diabetes-induced increase in AChE activity (p < 0.001), which was comparable to the standard donepezil hydrochloride treated group (Figure 3).

### Effect of *Murraya koenigii* extracts on rat brain histopathology

**Figure 4:**
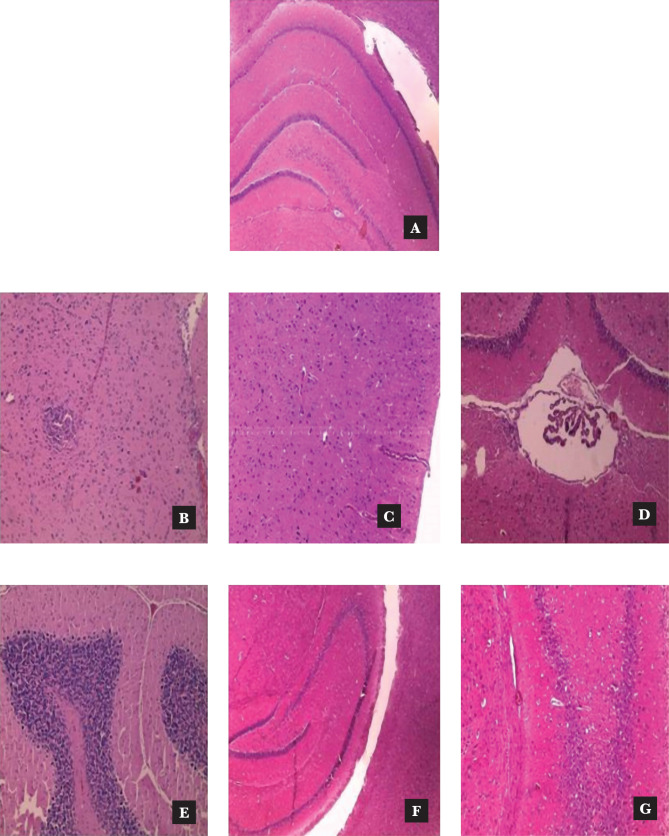
Effect of *Murraya koenigii* leaves extracts on the histological structure of the brain (cerebral cortex): A – The control group-it shows Dentate gyrus appeared normal. The normal cortex of cerebral hemispheres. Meninges appeared normal. B – Alloxan-induced diabetic group-it shows foci of necrosis noticed in the cerebral cortex region of the brain. C– Donepezil Hcl 2.5 mg/kg group shows frontal cortex appeared normal. D – *Murraya koenigii* ethanol 200mg/kg group shows mild degenerative changes noticed near the ventricles. E – *Murraya koenigii* ethanol 400 mg/kg group-it shows (a) Cerebellum appeared normal, (b) normal Purkinje cells, (c) normal granular layer, (d) white matter normal. F – *Murraya koenigii* Aq extract 200 mg/kg group-mild meningitis observed in the meningeal surrounding cerebral hemispheres. G – *Murraya koenigii* Aqueous extract 400 mg/kg-it showed dentate gyrus appeared normal. Tissues were stained with Hematoxylin and Eosin at magnification 100X.

**Figure 5:**
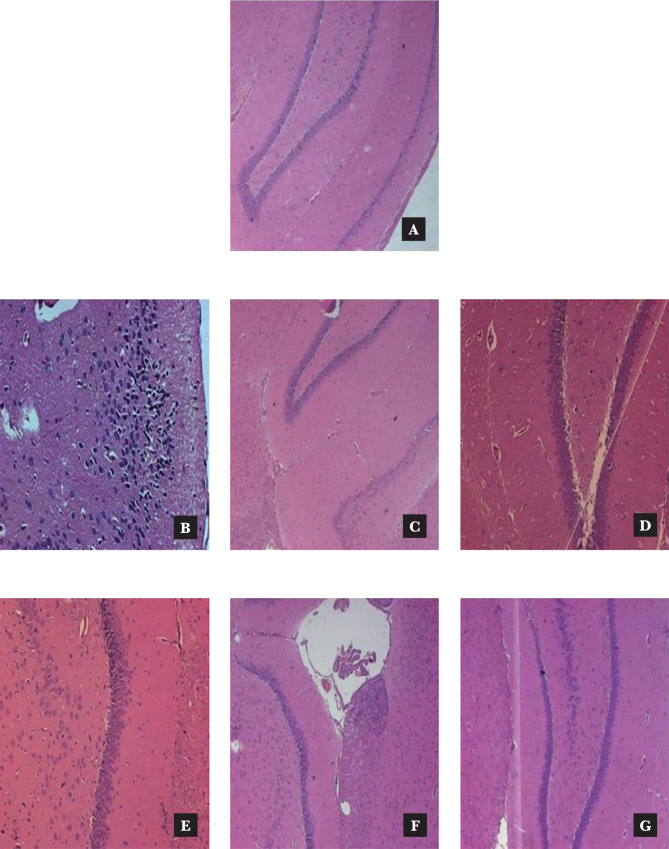
Effect of *Murraya koenigii* leaves extracts on the histological structure of the brain (hippocampus): A – The control group-it shows hippocampus appeared normal. B – Alloxan-induced diabetic group-foci of necrosis along with infiltration of inflammatory cells are noticed. C – Donepezil Hcl 2.5 mg/kg group-hippocampus appeared normal. D – *Murraya koenigii* ethanol 200 mg/kg group shows mild degenerative changes noticed near the ventricles. E – *Murraya koenigii* ethanol 400 mg/kg group Mild infiltration glial cells, normal hippocampus. F – *Murraya koenigii* Aq extract 200 mg/kg group-mild meningitis observed. G – *Murraya koenigii* Aqueous extract 400 mg/kg-normal ventricles of brain observed, normal hippocampus. Tissues were stained with Hematoxylin and Eosin at magnification 100X.

In normal control animals, no neuronal changes were observed whereas in Alloxan-induced diabetic rats, there was neurodegeneration and vacuolated cytoplasm. These changes were not observed in both the hippocampus or cerebral cortex regions in the *Murraya koenigii* extracts treated group (Figures 4 and 5).

## DISCUSSION

In this research, *Murraya koenigii* ethanolic and aqueous extracts were given to Alloxan-induced rats with cognitive dysfunction and oxidative stress for 30 days. The results demonstrate improved memory and learning. The movement of the animals when placed in a new chamber indicates spontaneous motor activity, and an increase in spontaneous motor activity indicates the level of CNS activity and hence the nootropic effect. In the Hole cross model, administration of the ethanolic and aqueous extracts increased the entries of a rat from a light area to a dark area. The fact that the frequency of entries has increased shows an enhancement in the learning and spatial memory of rats in comparison to Alloxan-treated cognitive deficit rats. Similar results were reported by Sherman *et al*. [29]. Ethanol extract showed a significant increase in the number of entries as compared to aqueous extract. The results are in correlation to the study conducted by Rao *et al*. Ethanolic extract showed 80% scavenging activity [30].

Open Field test is a valid model to screen the upgradation of learning and memory in rodent models as evidenced by improvement in movement activity. There was an increase in the number of squares crossed, indicating an enhancement of cognitive activity. Kishore *et al*. study on *Foeniculum vulgare* fruit extract in mice model revealed a significant increase in the number of squares crossed [31]. The Morris water maze is a reliable model for assessing memory and learning. Alloxan-induced diabetes resulted in a significant deterioration in cognitive performance. In comparison to normal control animals, there was an increase in latency to reach the hidden platform. The latency is a measurement of the signal's integration and association processes during the acquisition phase, and it is related to the time it takes for the input signal to reach the concerned brain structures (synaptic delays). *Murraya koenigii* ethanolic and aqueous extracts alleviated diabetic rats' cognitive deficits, resulting in a significant improvement in escape latency and a distinct increase in platform quadrant proportion and similar results reported by Biessels *et al*. [32].

The YM behavioral test is used to investigate short-term memory, general motor activity, and stereotypic behavior [33]. Spatial memory is studied by spontaneous alternation. In this test, the percentage of spontaneous alternation behavior was studied. A rise in the percentage spontaneous alternation behavior of *Murraya koenigii* ethanolic and aqueous extracts treated rats suggests an improved spatial long-term memory and learning as compared with the illness control group [34]. Ethanolic extract at 400mg/kg showed significant activity comparable to standard donepezil.

The results of this research showed notable improvement in spatial working memory, spatial working-reference memory, and spatial reference memory. This indicated a nootropic activity of *Murraya koenigii* ethanolic and aqueous extracts in diabetes-induced cognitive disability models. This activity may be attributed to the presence of phytoconstituents like flavonoids, anthocyanin glycosides, triterpenoids, and phytosterols. As oxidative stress increases, free radicals interact with neuronal cell membranes and cause lipid peroxidation, and there is increased production of TBARS. This increased lipid peroxidation end products accumulate in the neurons, causing degeneration and leading to Alzheimer's disease and cognitive deficit. There was an improvement in brain activity during treatment with *Murraya koenigii* ethanolic and aqueous extracts for 30 days, as evidenced by a decrease in lipid peroxidation activity (P < 0.001) [35]. Later complications of oxidative damage to various brain regions constitute morphological abnormalities and memory impairments. There was a decrease in GSH, GR, and CAT levels in the brains of diabetic-induced rats. Treatment with *Murraya koenigii* ethanolic and aqueous extracts significantly reduced the levels of TBARS and increased the GSH, GR, and CAT levels. The antioxidant properties of *Murraya koenigii* ethanolic and aqueous extracts might help to ameliorate cognitive dysfunction in diabetic animals. Similar findings were presented by Husna *et al*. [36].

The acetylcholinesterase levels are high in diabetics; ‘this enzyme hydrolyses acetylcholine present in the brain and results in cognitive decline’ [37]. There was a significant increase in acetylcholinesterase activity in the current study. Chronic administration of *Murraya koenigii* ethanolic and aqueous extracts prevented an increase in acetylcholinesterase activity. Hence, it can be inferred that chronic administration could prevent cognitive decline [38], cholinergic dysfunction, and reduction in oxidative injury in Alloxan-induced diabetic animals [39].

Notably, *Murraya koenigii* leaves ethanolic and aqueous extracts had successfully improved cholinesterase action in the brain. Nutriment consisting of *Murraya koenigii* leaves notably augment memory level and decreases cognitive impairment with dosage concentration stimulated by hyoscine and diazepam in juvenile and elderly female rats [40]. Further studies elucidating the clear mode of action and the safety characteristics of poly-phytonutrient of *Murraya koenigii* leaves are needed before the development of its botanical drug for the clinical indication of neuroprotection.

## CONCLUSION

In the present investigation, it can be inferred that chronic administration could prevent cognitive decline, cholinergic dysfunction, and reduction in oxidative injury in Alloxan-induced diabetic animals. Results are in accordance with the neurological justification for the traditional use of *Murraya koenigii* extracts in the treatment of neurodegenerative disorders, specifically Alzheimer's disease.

## Personal thanks

The authors are thankful to the authorities of Malla Reddy College of Pharmacy, Secunderabad, for providing support to this study.
